# Systems neuroscience in focus: from the human brain to the global brain?

**DOI:** 10.3389/fnsys.2015.00007

**Published:** 2015-02-06

**Authors:** Marios Kyriazis

**Affiliations:** ELPIs Foundation for Indefinite LifespansLondon, UK

**Keywords:** global brain, complex adaptive systems, human longevity, techno-cultural society, noeme, systems neuroscience

## Introduction

Human intelligence (i.e., the ability to consistently solve problems successfully) has evolved through the need to adapt to changing environments. This is not only true of our past but also of our present. Our brain faculties are becoming more sophisticated by cooperating and interacting with technology, specifically digital communication technology (Asaro, 2008).

When we consider the matter of brain function augmentation, we take it for granted that the issue refers to the human brain as a distinct organ. However, as we live in a complex technological society, it is now becoming clear that the issue is much more complicated. Individual brains cannot simply be considered in isolation, and their function is no longer localized or contained within the cranium, as we now know that information may be transmitted directly from one brain to another (Deadwyler et al., 2013; Pais-Vieira et al., 2013). This issue has been discussed in detail and attempts have been made to study the matter within a wider and more global context (Nicolelis and Laporta, 2011). Recent research in the field of brain to brain interfaces has provided the basis for further research and formation of new hypotheses in this respect (Grau et al., 2014; Rao et al., 2014). This concept of rudimentary “brain nets” may be expanded in a more global fashion, and within this framework, it is possible to envisage a much bigger and abstract “meta-entity” of inclusive and distributed capabilities, called the Global Brain (Mayer-Kress and Barczys, 1995; Heylighen and Bollen, 1996; Johnson et al., 1998; Helbing, 2011; Vidal, in press).

This entity reciprocally feeds information back to its components—the individual human brains. As a result, novel and hitherto unknown consequences may materialize such as, for instance, the emergence of rudimentary global “emotion” (Garcia and Tanase, 2013; Garcia et al., 2013; Kramera et al., 2014), and the appearance of decision-making faculties (Rodriguez et al., 2007). These characteristics may have direct impact upon our biology (Kyriazis, 2014a). This has been long discussed in futuristic and sociology literature (Engelbart, 1988), but now it also becomes more relevant to systems neuroscience partly because of the very promising research in brain-to-brain interfaces. The concept is grounded on scientific principles (Last, 2014a) and mathematical modeling (Heylighen et al., 2012).

## Augmenting brain function on a global scale

It can be argued that the continual enhancement of brain function in humans, i.e., the tendency to an increasing intellectual sophistication, broadly aligns well with the main direction of evolution (Steward, 2014). This tendency to an increasing intellectual sophistication also obeys Ashby's Law of Requisite Variety (Ashby, 1958) which essentially states that, for any system to be stable, the number of states of its control mechanisms must be greater than the number of states in the system being controlled. This means that, within an ever-increasing technological environment, we must continue to increase our brain function (mostly through using, or merging with, technology such as in the example of brain to brain communication mentioned above), in order to improve integration and maintain stability of the wider system. Several other authors (Maynard Smith and Szathmáry, 1997; Woolley et al., 2010; Last, 2014a) have expanded on this point, which seems to underpin our continual search for brain enrichment.

The tendency to enrich our brain is an innate characteristic of humans. We have been trying to augment our mental abilities, either intentionally or unintentionally, for millennia through the use of botanicals and custom-made medicaments, herbs and remedies, and, more recently, synthetic nootropics and improved ways to assimilate information. Many of these methods are not only useful in healthy people but are invaluable in age-related neurodegenerative disorders such as dementia and Parkinson's disease (Kumar and Khanum, 2012). Other neuroscience-based methods such as transcranial laser treatments and physical implants (such as neural dust nanoparticles) are useful in enhancing cognition and modulate other brain functions (Gonzalez-Lima and Barrett, 2014).

However, these approaches are limited to the biological human brain as a distinct agent. As shown by the increased research interest in brain to brain communication (Trimper et al., 2014), I argue that the issue of brain augmentation is now embracing a more global aspect. The reason is the continual developments in technology which are changing our society and culture (Long, 2010). Certain brain faculties that were originally evolved for solving practical physical problems have been co-opted and exapted for solving more abstract metaphors, making humans adopt a better position within a technological niche.

The line between human brain function and digital information technologies is progressively becoming indistinct and less well-defined. This blurring is possible through the development of new technologies which enable more efficient brain-computer interfaces (Pfurtscheller and Neuper, 2002), and recently, brain-to-brain interfaces (Grau et al., 2014).

**Figure 1:**
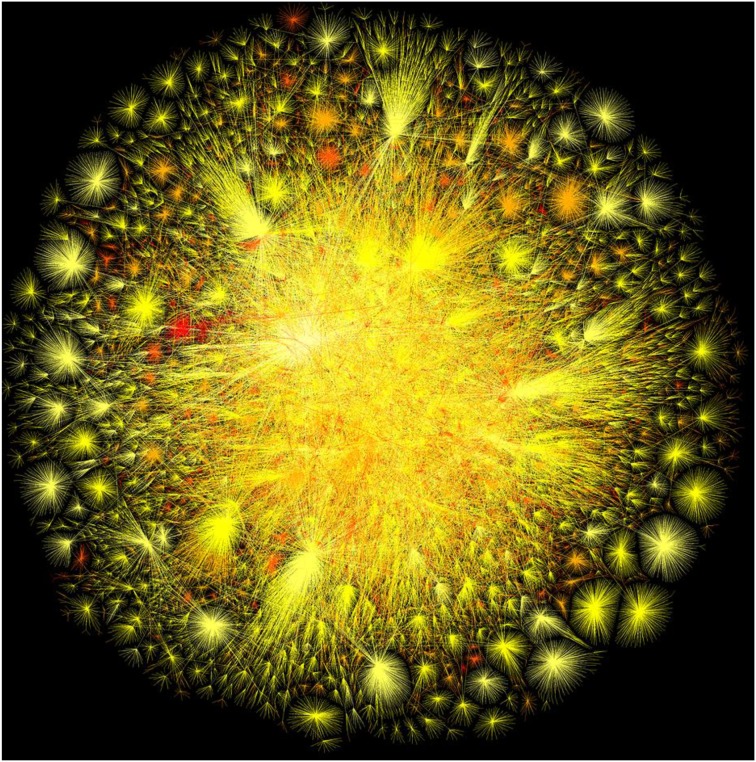
**Computer-generated image of internet connections world-wide (Global Brain)**. The conceptual similarities with the human brain are remarkable. Both networks exhibit a scale-free, fractal distribution, with some weakly-connected units, and some strongly-connected ones which are arranged in hubs of increasing functional complexity. This helps protect the constituents of the network against stresses. Both networks are “small worlds” which means that information can reach any given unit within the network by passing through only a small number of other units. This assists in the global propagation of information within the network, and gives each and every unit the functional **potential** to be directly connected to all others. *Source: The Opte Project/Barrett Lyon. Used under the Creative Commons Attribution-Non-Commercial 4.0 International License*.

We are now in a position expand on this emergent worldview and examine what trends of systems neuroscience are likely in the near-term future. Technology has been the main drive which brought us to the position we are in today (Henry, 2014). This position is the merging of the physical human brain abilities with virtual domains and automated web services (Kurzweil, 2009). Modern humans cannot purely be defined by their biological brain function. Instead, we are now becoming an amalgam of biological and virtual/digital characteristics, a discrete unit, or autonomous agent, forming part of a wider and more global entity (Figure 1).

## Large scale networks and the global brain

The Global Brain (Heylighen, 2007; Iandoli et al., 2009; Bernstein et al., 2012) is a self-organizing system which encompasses all those humans who are connected with communication technologies, as well as the emergent properties of these connections. Its intelligence and information-processing characteristics are distributed, in contrast to that of individuals whose intelligence is localized. Its characteristics emerge from the dynamic networks and global interactions between its individual agents. These individual agents are not merely the biological humans but are something more complex. In order to describe this relationship further, I have introduced the notion of the noeme, an emergent agent, which helps formalize the relationships involved (Kyriazis, 2014a). The noeme is a combination of a distinct physical brain function and that of an “outsourced” virtual one. It is the intellectual “networked presence” of an individual within the GB, a meaningful synergy between each individual human, their social interactions and artificial agents, globally connected to other noemes through digital communications technology (and, perhaps soon, through direct brain to brain interfaces). A comparison can be made with neurons which, as individual discrete agents, form part of the human brain. In this comparison, the noemes act as the individual, information-sharing discrete agents which form the GB (Gershenson, 2011). The modeling of noemes helps us define ourselves in a way that strengthens our rational presence in the digital world. By trying to enhance our information-sharing capabilities we become better integrated within the GB and so become a valuable component of it, encouraging mechanisms active in all complex adaptive systems to operate in a way that prolongs our retention within this system (Gershenson and Fernández, 2012), i.e., prolongs our biological lifespan (Kyriazis, 2014b; Last, 2014b).

## Discussion

This concept is a helpful way of interpreting the developing cognitive relationship between humans and artificial agents as we evolve and adapt to our changing technological environment. The concept of the noeme provides insights with regards to future problems and opportunities. For instance, the study of the function of the noeme may provide answers useful to biomedicine, by coopting laws applicable to any artificial intelligence medium and using these to enhance human health (Kyriazis, 2014a). Just as certain physical or pharmacological therapies for brain augmentation are useful in neurodegeneration in individuals, so global ways of brain enhancement are useful in a global sense, improving the function and adaptive capabilities of humanity as a whole. One way to augment global brain function is to increase the information content of our environment by constructing smart cities (Caragliu et al., 2009), expanding the notion of the Web of Things (Kamilaris et al., 2011), and by developing new concepts in educational domains (Veletsianos, 2010). This improves the information exchange between us and our surroundings and helps augment brain function, not just physically in individuals, but also virtually in society.

Practical ways for enhancing our noeme (i.e., our digital presence) include:

- Cultivate a robust social media base, in different forums.
- Aim for respect, esteem and value within your virtual environment.
- Increase the number of your connections both in virtual and in real terms.
- Stay consistently visible online.
- Share meaningful information that requires action.
- Avoid the use of meaningless, trivial or outdated platforms.
- Increase the unity of your connections by using only one (user)name for all online and physical platforms.

These methods can help increase information sharing and facilitate our integration within the GB (Kyriazis, 2014a). In a practical sense, these actions are easy to perform and can encompass a wide section of modern communities. Although the benefits of these actions are not well studied, nevertheless some initial findings appear promising (Griffiths, 2002; Granic et al., 2014).

## Concluding remarks

With regards to improving brain function, we are gradually moving away from the realms of science fiction and into the realms of reality (Kurzweil, 2005). It is now possible to suggest ways to enhance our brain function, based on novel concepts dependent not only on neuroscience but also on digital and other technology. The result of such augmentation does not only benefit the individual brain but can also improve all humanity in a more abstract sense. It improves human evolution and adaptation to new technological environments, and this, in turn, may have positive impact upon our health and thus longevity (Solman, 2012; Kyriazis, 2014c).

In a more philosophical sense, our progressive and distributed brain function amplification has begun to lead us toward attaining “god-like” characteristics (Heylighen, in press) particularly “omniscience” (through Google, Wikipedia, the semantic web, Massively Online Open Courses MOOCs—which dramatically enhance our knowledge base), and “omnipresence” (cloud and fog computing, Twitter, YouTube, Internet of Things, Internet of Everything). These are the result of the outsourcing of our brain capabilities to the cloud in a distributed and universal manner, which is an ideal global neural augmentation. The first steps have already been taken through brain to brain communication research. The concept of systems neuroscience is thus expanded to encompass not only the human nervous network but also a global network with societal and cultural elements.

### Conflict of interest statement

The author declares that the research was conducted in the absence of any commercial or financial relationships that could be construed as a potential conflict of interest.
